# Effects of Two Online Mindfulness-Based Interventions for Early Adolescents for Attentional, Emotional, and Behavioral Self-Regulation

**DOI:** 10.3390/pediatric16020022

**Published:** 2024-03-29

**Authors:** Bárbara Porter, Cristian Oyanadel, Ignacio Betancourt, Frank C. Worrell, Wenceslao Peñate

**Affiliations:** 1 Department of Psychology, Faculty of Social Sciences, Universidad de Concepción, Concepción 4030000, Chile; coyanadel@udec.cl (C.O.); ibetancourt@udec.cl (I.B.); 2 Berkeley School of Education, University of California, Berkeley, CA 94720-1670, USA; frankc@berkeley.edu; 3 Clinical Psychology, Psychobiology and Methodology School, Psychology Faculty, Guajara Campus, La Laguna University, 38200 Santa Cruz de Tenerife, Spain; wpenate@ull.edu.es

**Keywords:** mindfulness, childhood, adolescence, mental health, self-regulation, attention, emotional regulation, behavioral regulation, online MBIs

## Abstract

(1) Background: Mindfulness-based interventions (MBIs) have shown interesting preliminary effects on self-regulation processes in early adolescence. However, programs have typically combined different types of interventions with no understanding of the specific effect of each intervention type on attentional, emotional, and behavioral regulation. The objective of this research was to evaluate the effect of two MBIs—one focused on classic attentional practices and another focused on the recognition and expression of emotions—on attentional, emotional, and behavioral self-regulation in early adolescents. (2) Method: An experimental paradigm was used. A sample of 74 children aged between 8 and 12 years old were randomly assigned to three experimental conditions: (1) an MBI with a focus on attentional practices, (2) an MBI with a focus on recognition and expression of emotions, and (3) a control group. The interventions lasted 8 weeks, with a weekly, 1 h online synchronous session plus home practices. Children were evaluated before starting the intervention and at the end of the 8-week period. The assessed outcomes were (1) mindfulness; (2) emotional regulation; (3) attentional regulation, and (4) behavioral regulation. (3) Results: Children who participated in both intervention programs increased their mindfulness and emotional and behavioral regulation scores. Only children who participated in the MBI with a focus on attention showed significant changes in their ability to self-regulate attention. (4) Conclusions: The use of online MBIs, with attention to external and internal stimuli practices, can be a good strategy to strengthen self-regulation skills for attention, emotions, and behavior in early adolescence.

## 1. Introduction

Evidence suggests a high prevalence of mental disorders in youth [1,2], which can result in negative impacts on the quality of life in the short, medium, and long term [3,4,5,6]. The infrequent rate at which groups seek professional help increases the risk of failing to intervene in a timely manner, which has important consequences, since it makes it more difficult for youth to meet basic developmental milestones [7,8,9]. Thus, it is important to develop accessible and effective interventions aimed at preventing mental disorders and promoting mental health in children [10,11,12].

Specifically, early adolescence, that is, from 10 to 13 years of age [13,14,15,16], is a key stage in the development of self-regulatory processes, including attention, emotions, and behavior [17]. Self-regulation skills have been associated with higher levels of social and emotional well-being and a positive impact on academic functioning [14,15,18]. Young people with deficits in self-regulation skills are at greater risk for physical and mental pathologies in adulthood [3,19]. Strengthening self-regulation skills in early adolescence is related to improved physical and emotional health in adulthood, as well as greater social and economic achievements [17].

Mindfulness-based interventions (MBIs) are programmatically developed interventions focused on internal experiences, such as thoughts, feelings, or sensations, and the way an individual responds to them (i.e., acceptance vs. change) [20]. At the core of these interventions is mindfulness practice, which can be defined as an awareness of present experience with acceptance [21]. They have shown preliminary positive effects on self-regulatory processes in early adolescence, as well as decreases in depression, anxiety, and stress symptoms [22,23,24,25]. The strengthening of self-regulation skills, specifically in relation to attention [26,27], emotions [28,29], and behavior [30,31], have also been reported.

Although the extant literature shows promising results regarding the effects of MBIs, improving the specificity of the interventions and the quality of the research designs [23,32], as well as increasing the variety of modes to deliver interventions (e.g., face-to-face programs vs. programs carried out 100% online synchronously) [33], are challenges that need to be addressed. The wide diversity of mindfulness-based programs for children and youth and the heterogeneity of practices they include make it difficult to isolate the specific effects of certain practices on specific outcomes [23,33]. This information is crucial for developing more precise, effective, and accessible intervention programs. The objective of this research was to evaluate the effect of two MBIs conducted online—one focused on classic attentional practices and the other focused on the recognition and expression of emotions—on attentional, emotional, and behavioral self-regulation in early adolescents.

## 2. Method

An experimental design was used in the current study [34]. A sample of 74 children was randomly assigned to three conditions: (1) a mindfulness program with a focus on attention to external and internal stimuli, (2) a mindfulness program with a focus on the recognition and expression of emotions, and (3) a control group. The control group participated in one of the MBIs once the post-assessments were completed.

### 2.1. Participants

The sample consisted of boys and girls aged between 8 and 12 years old who were attending private, subsidized, and public schools in Chile. The distribution of the groups by gender and age is detailed in Table 1. Children with a current clinical diagnosis who were not receiving treatment, children with a cognitive or motor disability that prevented them from carrying out the evaluation tasks, and children who did not complete the initial evaluation were excluded from the study.

The sample size was determined a priori to conduct a repeated measures ANOVA with intra- and inter-group effects using the G*Power program version 3.1.9.2. Criteria included a medium effect size of 0.25, power of 0.8, an error rate of 5%, and three measurements per group, yielding a total sample of n = 36. Taking into consideration the probable dropout of some participants and missing data, a sample size of 72 boys and girls was recruited. We enrolled 74 participants but had to drop 4 participants who did not complete at least six of the eight sessions (75%), a minimum established in advance. The final sample for analysis consisted of 70 participants: 39 girls (55.71%) and 31 boys (44.29%). The assignment of the 70 participants to the three conditions is presented in Table 1.

To control the maximum number of participants in an intervention session, the 24 participants in the attention regulation intervention (see Table 1) were randomly assigned to two groups with 12 participants each. Similarly, the 25 participants in the emotion regulation intervention were randomly assigned to two groups, with 12 and 13 participants each. The intervention sessions were conducted online during the COVID-19 quarantine period, so there was no interaction with and among the participants apart from the intervention sessions.

### 2.2. Measures

Children were assessed before starting the intervention and at the end of the 8-week program. The variables assessed were the following: (1) mindfulness, evaluated with the Child and Adolescent Mindfulness Measure or CAMM [35]; (2) emotional regulation, evaluated with the Difficulties in Emotion Regulation Scale or DERS [36]; (3) behavioral regulation, assessed using the Behavior Rating Inventory of Executive Function or BRIEF-2 family form [37,38,39]; and (4) attention, using the flanker task [40,41].

#### 2.2.1. Child and Adolescent Mindfulness Measure, CAMM [35]

The Child and Adolescence Mindful Measure (CAMM) is a self-report instrument that evaluates trait mindfulness in children and adolescents aged between 9 and 18 years old [42], and CAMM scores have been validated in Chile [35]. The ten-item CAMM assesses mindfulness as a unidimensional construct, defined as present-focused awareness and the ability to be non-judgmental about internal experiences [42]. It uses a five-point Likert scale ranging from 0 (never) to 4 (always). A validation study conducted in Chile and Spain included a sample of 2113 Chilean (n = 307 children; n = 687 adolescents) and Spanish (n = 490 children; n = 629 adolescents) youth. A confirmatory factor analysis (CFA) revealed that the factor coefficients for three items were very low in all samples. These items were eliminated, resulting in a new seven-item version of the CAMM, which was supported by the CFA. Scores on the seven-item CAMM yielded a reliability estimate of 0.67 for Chilean children (aged 8 to 12 years old) and 0.85 for Chilean adolescents (aged 13 to 19 years old) [35]. Questions on the CAMM are phrased negatively (e.g., “I think that some feelings I have are bad and I should not have them”), so a decrease in the score indicates an increase in the dispositional mindfulness variable. Higher scores indicate lower dispositional mindfulness. The average score of this test for 10-year-old Hispanic children is 18 (SD = 4.8) [43,44].

#### 2.2.2. Difficulties in Emotion Regulation Scale, DERS [36]

The DERS is a self-report measure that, in its original version for adults, consists of 36 items [45]. It was designed to evaluate six emotional dysregulation dimensions: (1) difficulties in recognizing emotional responses, (2) difficulties in clarifying emotional responses, (3) difficulties controlling impulsive behavior facing negative emotions, (4) difficulties engaging in goal-oriented behaviors facing negative emotions, (5) non-acceptance of negative emotional responses, and (6) limited access to effective emotional regulation strategies. In adult samples, DERS scores have yielded an internal consistency estimate of 0.93 and a test–retest reliability estimate of 0.88. The internal consistency for subscale scores has ranged from 0.80 to 0.89 [45]. In the present study, we used the version validated in Chile, which consists of 25 items that load onto five factors. The internal consistency of subscale scores in Chile ranged from 0.69 to 0.89, with a reliability estimate of 0.92 for the total score in two samples [36]. A version of the DERS adapted for adolescents in which some words were modified based on cognitive interviews and the opinion of expert judges was used in the current study [46]. Items 4, 5, 8, 10, 14, 24, and 25 were modified slightly to make them easier to understand. For example, Item 14 (“When I feel upset, I find it difficult to think about anything else”) was changed to “When I feel bad, I find it difficult to focus on other things.” As this instrument evaluates difficulties in emotional regulation, a decrease in the score indicates an improvement in skills. A higher score will indicate emotional dysregulation. The cutoff score of this test for Chilean adolescents is 73 [36].

#### 2.2.3. Behavior Rating Inventory of Executive Function, BRIEF-2 Family Form [37,38]

This instrument evaluates executive functions aimed at guiding and organizing cognition, emotion, and behavior in children and adolescents aged between 5 and 18 years old. There is a family version (for caregivers), and another for teachers. In the present study, the family version was used. The family version contains 86 items that assess various behaviors, which are rated on a Likert scale from 1 to 3 (1 = never, 3 = always). When responding, caregivers are asked to think about behaviors observed in their children during the last month. The BRIEF-2 has nine subscales: (1) inhibition, (2) flexibility, (3) emotional control, (4) initiative, (5) working memory, (6) planning, (7) materials organization, (8) task supervision, and (9) self-monitoring. In the present study, the Global Executive Function Index, which is based on all subscales, was used. Scores on the BRIEF-2 family version have yielded a test–retest reliability estimate of 0.82 [37,38]. In its Chilean adaptation [39], reliability estimates for scores on the nine subscales were as follows: (1) flexibility, 0.89; (2) emotional control, 0.94; (3) initiative, 0.87; (4) working memory, 0.94; (5) planning, 0.91; (6) organization of materials, 0.92; (7) task supervision 0.88; (8) inhibition, 0.95; and (9) self-monitoring, 0.85. Questions on the BRIEF-2 are phrased in a negative way (e.g., “He has explosions of anger”), so a decrease in the score indicates an increase in behavioral self-regulation.

#### 2.2.4. Flanker Task [40,41]

The flanker task is a computerized test designed in 1970 by Eriksen and Eriksen. It assesses the components of the tripartite model of attention, which is consistent with the theorizing of Petersen and Posner [47]: alertness (state of vigilance and preparation to respond to environmental stimuli), orientation (ability to direct and limit attention to a specific stimulus), and conflict monitoring (prioritizing the localization of attention between competing stimuli) [26]. The test has four sections: a sample test to ensure that the child understands the instructions, followed by three subsequent tests. Respondents are instructed to observe a screen on which five letters appear simultaneously. The person must respond based on the middle letter. If the middle letter corresponds to the letters X or C, the person must press A on the keyboard. If the middle letter on the screen corresponds to the letters V or B, the person must press the L key. The middle letter can be surrounded by similar letters—that is, a congruent stimulus, for example, BBBBB—or it can be surrounded by different letters—that is, an incongruent stimulus, for example, XXBXX. The time it takes to respond and the accuracy of the response can be affected by the congruence of the stimulus, as relevant information must be attended to and irrelevant information ignored [40,41]. In the present study, the flanker task was administered using the PsyToolKit platform https://www.psytoolkit.org/experiment-library/flanker.html#_introduction (accessed 5 January 2024).

This test provides four attention self-regulation scores: (1) response time to a congruent stimulus, (2) response time to an incongruent stimulus, (3) the percentage of errors in responses to congruent stimuli, and (4) the percentage of errors in responses to incongruent stimuli. In the present study, we used the percentage of errors in response to incongruent stimuli as a measure of attention, since this measure allows us to evaluate the three components of attention relevant to this study, that is, alertness, orientation, and conflict monitoring [27,48].

### 2.3. Interventions

The three treatment groups were the independent variables: (1) treatment based on mindfulness with a focus on the regulation of attention, (2) treatment based on mindfulness with a focus on the regulation of emotions, and (3) a passive control group. The design of both intervention programs was carried out with the supervision of two independent experts, who evaluated the exercises included in each program based on their relevance to developing the regulation of attention, emotions, and behavior.

Intervention instructors were first selected and then trained. Recruitment was based on two criteria: (1) people with verifiable mindfulness training and (2) people with experience working with children. The three instructors who were selected met these criteria. One of them was a child and adolescent psychiatrist, another was a child and adolescent psychologist, and the third was a primary teacher. They participated in the Instructor Training Program, which consisted of eight sessions of 2 h each. This program included eight in-person 1 h sessions on all the practices for children included in both interventions, followed by an additional hour on the theoretical and empirical foundations of mindfulness. A manual detailing the curriculum for each session was prepared for the instructors to use in delivering the intervention sessions to ensure fidelity to the design.

Before starting the interventions, a manual and a box of materials were sent by mail to each student participant, as well as a set of audio recordings with exercises to practice between sessions. The maximum number per group was 13 children, with two instructors per group. The lead researcher was one of the instructors, and one of the three trained instructors served as a co-instructor in each session. The lead instructor met the criteria for instructors, as she had previous certified training in both MBI for adults and children. For the sessions, a Google Meet link, through which the boys and girls accessed the synchronous session from their homes, was sent weekly to the email of each parent.

#### 2.3.1. Intervention 1: Program Based on Mindfulness with a Focus on Attention

The exercises included in the attention curriculum were aimed at developing attention to both external stimuli (e.g., sounds, flavors, and colors) and internal stimuli (e.g., sensations, emotions, thoughts, and breathing). This intervention lasted 8 weeks with a single 1 h session per week. The name given to this attention-focused program was “Monkey Mind, Where Are You?” A summary of the exercises included in each session is provided in Table 2.

#### 2.3.2. Intervention 2: Mindfulness-Based Program with a Focus on Emotions

The exercises in the emotion-focused program were aimed at developing emotional regulation, such as observing and identifying emotions in the body, describing these emotions, observing and describing thoughts associated with emotions, and describing behaviors that arise with emotions. This program also had exercises to develop empathy and compassion. The program lasted 8 weeks with a single 1 h session per week. This intervention was also manualized, with exercises to do at home, supported by audio recordings and concrete material. The name given to this program was “I am a dragon, what can I do with my fire?” A summary of the program curriculum is detailed in Table 3.

Each intervention curriculum was assessed and approved by two experts external to the research team. The first two sessions and the eighth session in each program are similar in content. Sessions 3 to 7 are different. The first two sessions introduce the basic aspects of mindfulness practice (understanding its meaning and performing basic body and breathing practices) before moving into practices focused on attentional regulation vs. emotional regulation. Session 8 involves bringing closure to the training program.

### 2.4. Procedure

**Figure 1:**
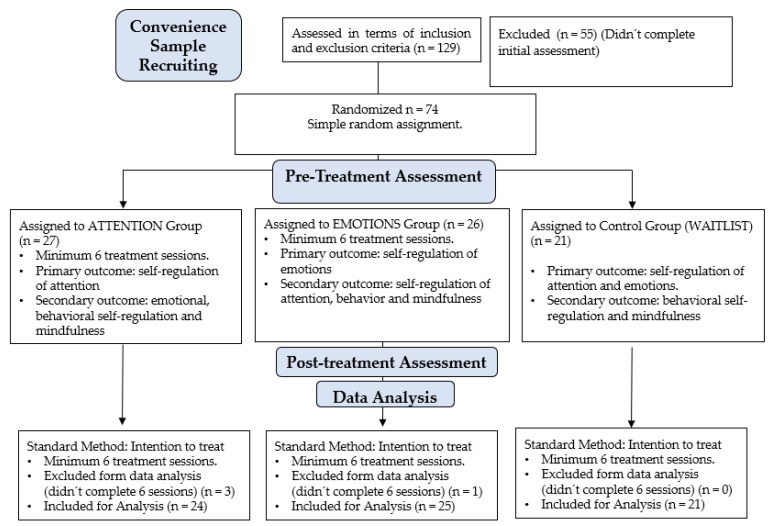
Flowchart.

Participants were a non-random, convenience sample. The recruitment was done amid the COVID-19 pandemic through social networks. Once contact was established with the caregivers, information was sent via email. Selection was based on the sociodemographic information provided and the exclusion criteria. Subsequently, participants were randomly assigned to each of the three study conditions. The initial equivalence of the groups in terms of gender, age, and type of schools (private, public, or subsidized) was ensured. This process is summarized in Figure 1.

#### Data Analysis

The analysis of the results began with reporting the main descriptive statistics for the variables. To answer the research questions, linear mixed models were used, considering the treatment condition and time as fixed effects; a random intercept was considered for each subject. This analysis was selected as it allows for correlations within clusters with common characteristics [49], in this case, each study participant. To analyze the influence of the factors, F tests were used to test the significance of each fixed effect in the total model [50]. The Satterthwaite approximation for obtaining degrees of freedom was used, controlling for age and gender to ensure that any significant effects were due to the factors of interest (i.e., treatment or time).

The interaction effects between time and treatment were analyzed. If an interaction effect was significant, the marginal means at each level were analyzed to explore the differences in more detail, using the Bonferroni adjustment to control for Type I error. If no interaction effect was found, the main effects for time and treatment condition were examined. Analyses were run using the R program, version 4.1.0. We report both statistical significance and Hedges’ g effect sizes.

## 3. Results

**Figure 2:**
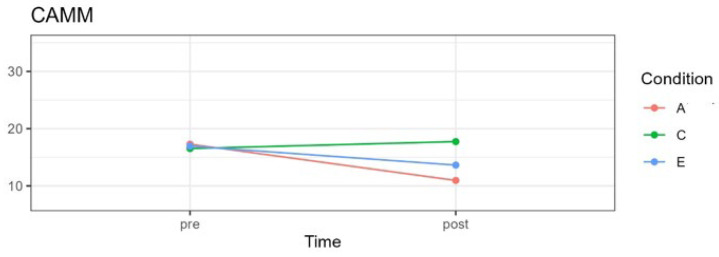
CAMM mean scores for three experimental conditions in pre- and post-treatment. A = attention group; C = control group; E = emotion group.

**Figure 3:**
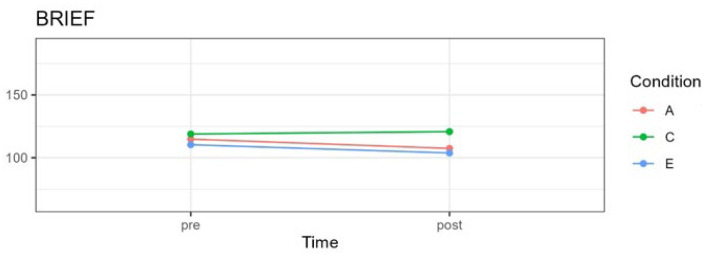
BRIEF-2 family version mean scores graph for three experimental conditions at pre- and post-treatment. A = attention group; C = control group; E = emotion group.

**Figure 4:**
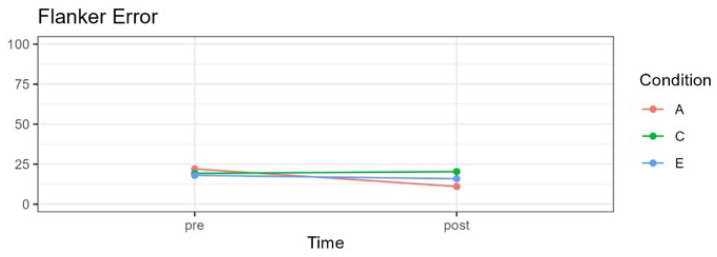
Mean flanker task scores percentage of errors for incongruent stimuli for three experimental conditions in pre- and post-treatment. A = attention group; C = control group; E = emotion group.

**Figure 5:**
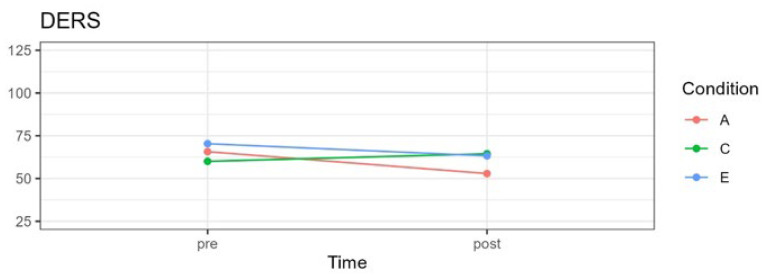
DERS mean scores for three experimental conditions in pre- and post-treatment. A = attention group; C = control group; E = emotion group.

Results are summarized in Table 4 and Figure 2, Figure 3, Figure 4 and Figure 5 and described below for each of the outcome variables.

### 3.1. Mindfulness

A significant interaction effect between time and treatment was observed for mindfulness, *F*(2;59.21) = 24.64, *p* < 0.001, in addition to a significant main effect for time, *F*(1;65.58) = 37.54, *p* < 0.001. The main effect for the three experimental conditions was not significant, *F*(1;59.92) = 2.53, *p* = 0.08. Figure 2 shows the means for each condition pre- and post-treatment.

No significant differences were observed between the three experimental groups pre-treatment, comparing the attention with the control condition, *t*(85.3) = 0.461, *p* > 0.99; attention with the emotion condition *t*(84.8) = 0.186, *p* > 0.99; and emotion with the control condition, *t*(85.1) = −0.275, *p* > 0.99. However, from pre-treatment to post-treatment, significant differences were observed for the attention condition, *t*(65.4) = 8.16, *p* < 0.0001, g = −1.28, and the emotion condition, *t*(65.8) = 4.05, *p* = 0.0001, g = −0.68. These differences are evident in the mean scores shown in Table 4. For the control group, there was no significant difference between pre- and post-treatment, *t*(63.1) = −1.50, *p* = 0.137, g = 0.27, with the post-treatment mean showing a slight increase.

### 3.2. Behavioral Self-Regulation

A significant interaction effect of time and condition for behavioral self-regulation was observed, *F*(2;58.29) = 3.88, *p* = 0.026, in addition to a significant main effect for time, *F*(1;66.34) = 5.83, *p* = 0.018. However, the experimental condition did not result in statistical significance for behavioral self-regulation, *F*(2;59.22) = 2.09, *p* = 0.13. Figure 3 shows the means on BRIEF-2 scores for each condition pre- and post-treatment.

No significant differences were observed between the three experimental groups pre-treatment, comparing the attention with the control condition, *t*(78.0) = −0.488, *p* > 0.99; the attention with the emotion condition *t*(76.5) = 0.771, *p* > 0.99; and the emotion with the control condition *t*(77.5) = 1.221, *p* = 0.676. For the attention group, a significant pre- to post-treatment effect was observed, *t*(66.3) = 2.72, *p* = 0.0083, g = 0.34. A statistically significant difference for pre- vs. post-treatment was also found for the emotion group, *t*(66.7) = 2.27, *p* = 0.026, g = −0.38. For both experimental conditions, a decrease in scores from pre- to post-treatment is evident in Table 4 and Figure 3. In the control group, there were no significant changes, t (63.4) = −0.78, *p* = 0.437, g = 0.10, indicating similar mean scores on behavioral self-regulation at both time points.

### 3.3. Attention Self-Regulation

Self-regulation of attention was evaluated using the computerized flanker task, which assesses the percentage error in responses to incongruent stimuli. A significant interaction effect was observed between time and condition, *F*(2;57.26) = 3.98, *p* = 0.023. However, the main effects for time, *F*(1;61.36) = 3.27, *p* = 0.075, and experimental condition, *F*(2;58.13) = 0.07, *p* = 0.930, were not statistically significant.

Figure 4 shows changes in the percentage of errors when faced with incongruent stimuli, evaluated using the flanker test.

No significant differences were observed among the three experimental groups pre-treatment, comparing the attention with the control condition, *t*(98.4) = 0.933, *p* > 0.99; the attention with the emotion condition *t*(99.3) = 0.908, *p* > 0.99; and the emotion with the control condition *t*(98.8) = −0.057, *p* > 0.99. A significant effect was observed pre- to post-treatment only for the attention group, *t*(63.4) = 3.271, *p* = 0.0017, g = −0.87. For both the emotion and control groups, there were no significant differences: *t*(62.4) = 0.373, *p* = 0.710, g = −0.13, and *t*(60.8) = −0.456, *p* = 0.650, g = 0.07, respectively. This finding is also evident in the means in Table 4.

### 3.4. Emotional Self-Regulation

A significant interaction effect was observed between time and treatment for DERS scores, *F*(2;57.81) = 10.57, *p* < 0.001, as well as a significant main effect for time, *F*(1;63.13) = 12.38, *p* < 0.001. There was no main effect for the experimental condition, *F*(2;58.99) = 2.14, *p* = 0.126. Figure 5 shows the changes in emotional self-regulation over time.

No significant differences were observed between the three experimental groups pre-treatment, comparing the attention with the control condition, *t*(92.6) = 1.162, *p* = 0.745; the attention with the emotion condition *t*(91.2) = −1.116, *p* > 0.802; and the emotion with the control condition *t*(92.6) = −2.206, *p* = 0.089. Significant differences were observed between pre- and post-treatment for the attention condition, *t*(63.8) = 4.99, *p* < 0.0001, g = −1.03, and the emotion condition, *t*(64.0) = 2.58 *p* = 0.012, g = −0.53, which resulted in decreases their mean scores (see Figure 5 and Table 4). The control group’s scores did not change significantly from pre- to post-treatment (g = 0.30), although the mean increased slightly.

## 4. Discussion

The aim of the present study was to evaluate the effects of two programs based on mindfulness—one with a focus on attention and the other with a focus on emotions—on the levels of mindfulness and self-regulation of attention, emotions, and behavior in boys and girls aged between 8 and 12 years old. Results suggest that children who participated both in the attention-focused program and the emotion-focused program increased their mindfulness and behavioral and emotional regulation scores. However, only children who participated in the program with a focus on attention seemed to improve their ability to regulate attention, showing a significant decrease in the percentage of errors made in response to incongruent stimuli. Children who participated in the group with a focus on emotions and those in the control group maintained their error percentage rates.

### 4.1. Mindfulness

Results suggest that both programs favor mindfulness skill development. This finding is consistent with previous studies on the effects of basic body attention and breathing exercises on trait mindfulness in the child and adolescent population [33,51]. Likewise, the results are consistent with the evidence related to the structure of the program, specifically its duration and frequency. In this case, both programs lasted 8 weeks with a weekly session of 1 hour, a decision that was made based on the effectiveness of existing models [52,53,54,55,56,57]. Based on the results obtained, we suggest that the proposed structure (8 weeks, weekly frequency, and 60 min sessions), in addition to the inclusion of the core mindfulness practices detailed in each curriculum, can be effective for mindfulness development in youth.

### 4.2. Behavioral Self-Regulation

Results show an increase in behavioral self-regulation skills for the children who participated in both the program with a focus on attention and the program with a focus on emotions, with no changes in these skills for the control group. This ability was evaluated by parents and caregivers using the BRIEF-2 family version [39,58,59]. For the analysis, the Global Index of Executive Function, which is a score that summarizes the nine clinical scales of the BRIEF-2 (inhibition, flexibility, emotional control, initiative, working memory, planning and organization, self-monitoring, supervision of your task, and organization of materials), was used. The increase observed in these skill sets by parents of the participating children is consistent with previous studies. Research points to the positive effects of practicing mindfulness on executive functions in children and adolescents [60,61,62], specifically on response inhibition [63] and self-monitoring [64]. However, there is some debate in the literature, as not all mindfulness-based programs produce significant changes in these skills [65]. Based on this study, we propose that the formula of eight 60 min sessions once a week that include basic mindfulness exercises can help increase behavioral self-regulation of boys and girls aged from 8 to 12 years old. Future studies will be needed to determine the specific exercises and practice doses required to produce changes in these skills at other ages and stages of development.

### 4.3. Attentional Self-Regulation

Results suggest that only children exposed to the program with a focus on attention improved self-regulation of attention, showing a decrease in the percentage of errors made in response to incongruent stimuli. The children who participated in the program with a focus on emotions and those who participated in the control group did not show either statistically or practically significant changes on this indicator. A possible explanation is related to the types of practices contained in each program. In the program with a focus on attention, all eight sessions included classic mindfulness practices, called contemplative practices (such as conscious breathing, conscious movement, sitting meditation, and body scan). In the program with a focus on emotions, these classic mindfulness practices are included in three sessions (1, 2, and 8), and the other sessions include practices focused on the awareness and expression of emotions and generative practices. The latter seeks to generate specific states such as empathy or compassion [66,67,68,69,70], which do not result in increased attention based on our findings. Nevertheless, it should be noted that the percentage of errors for incongruent stimuli is only one indicator of attention self-regulation, and this finding should be replicated using other measures of attention in future studies.

### 4.4. Emotional Self-Regulation

Results suggest that children who participated in the program with a focus on attention and the children who participated in the program with a focus on emotions improved their emotional regulation scores, unlike the children in the control group. The fact that children who participated in the program that focused solely on attention improved their emotional self-regulation skills is interesting to consider.

Self-regulation is defined as the ability to monitor and control one’s cognitions, emotions, and behavior in pursuit of goal achievement, or alternatively, the ability to adapt to the cognitive and emotional demands of specific situations [71]. These skills should be interrelated, consistent with the model proposed by Tang and Hölzel [27], and the findings showcase the interrelationships among attentional and emotional regulation and self-awareness. In the case of attentional regulation, there is evidence indicating that it is a critical component of self-regulation, specifically in early adolescence [71,72]. Attentional processes play an important role in self-regulated action and may be especially important in the regulation of emotions in infants, children, and adolescents [14,15]. Thus, the results of this study show the impact of strengthening attentional regulation as a foundation for facilitating emotional regulation during this critical developmental period [18,27,48,73]. These findings suggest that the attention-based intervention can be chosen as the first option, as it affects emotional, attentional, and behavioral self-regulation skills. On the other hand, the emotion-based program could be more appropriate for youth who already have well-developed attentional self-regulation skills but have difficulties in emotion regulation. This intervention may also be appropriate for clinical populations with mood disorders. The present study was carried out with a non-clinical sample; therefore, these questions will need to be addressed in future research.

The development of self-regulation skills in early adolescence is associated with better mental health in adulthood [17]. Developing low-cost interventions that are accessible to a greater number of adolescents can be a good way to prevent mental health problems in this population. The online mode can also improve access to adolescents who, due to distance or cost, cannot access in-person MBIs.

### 4.5. Limitations

The present study has both methodological and conceptual limitations. Regarding methodological limitations, it is important to point out the context. The COVID-19 pandemic involved a series of difficulties related to the confinement of the quarantines that lasted for months in our country. Thus, this situation forced us to change the way we intervene and evaluate children. In typical times, the intervention would have been carried out in schools face-to-face but had to be modified to be carried out online. The online modality can be considered a strength as it can improve the accessibility of this type of intervention, as was mentioned previously. However, this modality also had some limitations. First, the online administration of the evaluation and self-assessment measures could have been affected by the children’s reading comprehension. Although an analysis was conducted on the level of the language of the measures, and we asked parents to be available to help children who needed assistance, we did not have direct control over this issue. Second, the sample in the present study was small and non-random, being recruited from networks available during the pandemic. Therefore, the external validity of the results is decreased. It will be necessary to carry out subsequent studies with larger samples. Third, a longer-term follow-up assessment, for example, 2 or 3 months after finishing the intervention, could be included in future studies to evaluate if the gains in mindfulness and self-regulatory skills are maintained or if these are lost over time. It is important to examine this outcome to determine the duration of the interventions’ effects, as it may be necessary to carry out new interventions from time to time to maintain the benefits.

### 4.6. Future Research

Future studies need to be conducted to generate accessible and lower-cost proposals for child and adolescent samples and to adjust interventions for specific groups.

Regarding generalization, we need to move toward preventive and universal models [74,75,76]. Given the present study, this possibility is not limited to face-to-face interventions, but there is preliminary evidence of the effects of programs taught 100% online. This finding opens up the possibility of universalizing these types of practices, given that most interventions for this population continue to be exclusively face-to-face [33]. Future research could investigate the effects of various modalities: 100% in-person, hybrid modality, and 100% synchronous online modality. This study also suggests investigating the effect of e-learning modalities via the gamification that has already been introduced in the educational field [77,78,79], or the use of immersive practice technologies that can make mindfulness closer and more accessible, which has already been incorporated into the clinical context [80]. Future research can also focus on maximizing the feasibility of the intervention, considering aspects such as the frequency and duration of the sessions, adherence to the program both in the sessions and outside of them, and parental involvement, among others.

The present study was carried out mainly on a non-clinical sample. It will be necessary to examine the effects of these types of programs on children with diagnoses related to neurodevelopmental disorders (such as ADHD, ASD, and SLI), as well as boys and girls with internalizing (e.g., anxious or depressed) and externalizing (e.g., oppositional defiant disorder) symptoms [81,82]. It will also be necessary to adapt these types of interventions for children with disabilities, such as children who are blind or deaf or have permanent motor difficulties, and evaluate the programs’ effects, both with respect to self-regulation and mental health skills, questions which have been investigated in adult samples [83,84,85].

It will also be important to evaluate the programs’ effects on younger children and older adolescents. A conceptual definition of mindfulness that can be operationalized for every period and provide structure in the development of mindfulness interventions throughout the life cycle is needed [33]. Such a definition will be crucial in avoiding interventions that are atomized and disconnected from each other.

## 5. Conclusions

The present study was designed to assess the effectiveness of two intervention programs based on mindfulness on trait mindfulness and self-regulation of attention, emotions, and behavior in boys and girls aged between 8 and 12 years old.

The main conclusions of the present study are presented below:

(1) Results show that both interventions resulted in improvements in trait or dispositional mindfulness.
(2) Both the program with a focus on attention and the program with a focus on emotions resulted in significant changes in the regulation of emotions and behavior.
(3) Only children who participated in the program with a focus on attention showed a significant change in the precision of their responses, decreasing the percentage of errors for incompatible stimuli. The program with a focus on emotion did not affect attentional self-regulation.
(4) Both programs examined in this study contributed to strengthening self-regulation skills of emotions and behavior in boys and girls aged between 8 and 12 years old, with the program focusing on attention also being effective in terms of attentional self-regulation.

Based on these results, we suggest that online programs can be effective in developing self-regulation skills in adolescents. The detailed manualization of the interventions and the online modality will facilitate replicating this study with minimal implementation costs. We suggest that future studies address the limitations we described above, both to replicate the core findings and to extend them to other groups.

## Figures and Tables

**Table 1:** Assignment of participants to conditions by age and gender.

	Girls		Boys			
Group	n	Age (SD)	n	Age (SD)	Total N	Average Age
Attention	14	9.66 (1.33)	10	9.90 (0.88)	24	9.91
Emotions	13	10.00 (0.60)	12	9.50 (0.80)	25	9.75
Control	12	9.08 (1.04)	9	9.11 (1.05)	21	9.09
Total	39	9.66 (1.10)	31	9.51 (0.92)	70	9.60

**Table 2:** Program with a focus on attention, “Monkey Mind, Where Are You?”: summary of practices and materials per session.

Session	Mindfulness Practices	Materials
1	Attention to the sound of the bell Attention to the body in motion (seaweed) Attention to breathing (bubble breathing)	Bubble bottle Audio to perform breathing exercise at home “Breathing Bubbles”
2	Attention to the sound of the bell Preparation and practice with the calmness jar. Mindful breathing (locate the breath in the nose, ribs, abdomen)	Calmness jar
3	Attention to the sound of the bell Attention to the body in motion (funny walk) Attention to the body at rest (creation of jewelry with plasticine and body scanner)	Plasticine Audio to practice body scanning at home
4	Attention to the sound of the bell Attention to the body in movement (standing yoga) Mindful breathing (breathing with stones at the bottom of the sea)	Colored stones Audio to practice breathing exercise at home
5	Attention to the sound of the bell Attention to the five senses Attention to the body at rest and mindful breathing (floating like an otter)	Mandala, colored pencils, reflective cardboard, pen, sandpaper, and fruit to exercise the five senses Audio to practice exercise attention to the body at rest and breathing
6	Attention to the sound of the bell Attention to the body in movement (synchronized dance) Attention to breathing and thoughts (the comic of thoughts)	Comic drawing of thoughts Audio to practice exercise of attention to breathing and thoughts
7	Attention to the sound of the bell. Attention to the body in motion (creating the great storm) Breathing and visualization exercise (my favorite calm place)	Colored pencils Audio to practice breathing attention and visualization exercise
8	Attention to the sound of the bell. Attention to the body in motion (participants choose from those carried out in the program) Mindful breathing and visualization exercise (the good gardener)	Seeds

**Table 3:** Program with focus on emotions “I am a dragon, what can I do with my fire?”: summary of practices and materials per session.

Session	Mindfulness Practices	Materials
1	Attention to the sound of the bell. Attention to the body in motion (seaweed) Attention to breathing (bubble breathing)	Bubble bottle Audio to perform breathing exercise at home “Breathing Bubbles”
2	Attention to emotions in the body Breathing practice with the calmness jar Calendar of happy moments	Calmness jar. Happy moments calendar.
3	Attention to the body in motion (emotions walk) Attention to the five senses: creating the dragon with plasticine. Attention to the body at rest (body ccanner focused on emotions)	Plasticine Body scan focused on emotions audio.
4	Attention to the body in movement (dance of emotions) Practice of emotions (body scan focused on a present emotion and visual expression of what is perceived) Mindful drawing of emotions	Colored pencils Awareness of emotions practice audio
5	Attention to the intensity of emotions with the “thermometer” of emotions Wave of emotions: attention to the increase and decrease of emotions Attention to the body at rest and mindful breathing	Thermometer and wave of emotions Mindful breathing with emotions audio
6	Attention to the body in motion (storm of emotions) Attention inwards: my inner emotional climate Mindful breathing and attention to five senses to calm emotions	Mandala, colored pencils, reflective cardboard, pen, sandpaper, and fruit to exercise the five senses
7	Attention to the body in motion Mindful breathing and attention to difficult emotions. Visualization exercise (my favorite calm place)	Colored pencils Visualization practice audio
8	Attention to the sound of the bell. Attention to the body in motion (participants choose from those carried out in the program) Mindful breathing and visualization exercise (the good gardener)	Seeds

**Table 4:** Means and (SD) of the variables according to experimental group, by time and condition.

		Pre-Treatment Time			Post-Treatment Time		
Variables	Tests	Attention	Emotion	Control	Attention	Emotion	Control
Mindfulness	CAMM *	17.28	16.94	16.52	10.95	13.63	17.73
		(5.81)	(4.93)	(3.76)	(3.65)	(4.69)	(4.86)
Behavior self-regulation	BRIEF-2	114.81	110.36	118.89	107.42	103.78	120.78
		(22.91)	(18.32)	(21.02)	(19.68)	(15.66)	(17.88)
Attention self-regulation	Flanker test	22.02	18.00	19.25	11.04	15.87	20.33
		(14.79)	(16.50)	(14.07)	(9.44)	(16.74)	(15.96)
Emotion self-regulation	DERS	65.71	70.36	60.05	52.90	63.26	64.47
		(15.06)	(12.46)	(11.64)	(8.67)	(14.12)	(17.06)

* CAMM: Child and Adolescent Mindfulness Measure/BRIEF-2: Behavioral Rating of Executive Function-2 Family Form/DERS: Difficulties in Emotional Regulation Scale.

## Data Availability

Please contact the corresponding author to get the data.
